# Simulation of angiogenesis in three dimensions: Application to cerebral cortex

**DOI:** 10.1371/journal.pcbi.1009164

**Published:** 2021-06-25

**Authors:** Jonathan P. Alberding, Timothy W. Secomb

**Affiliations:** 1 BIO5 Institute, University of Arizona, Tucson, Arizona, United States of America; 2 Department of Physiology, University of Arizona, Tucson, Arizona, United States of America; Oxford, UNITED KINGDOM

## Abstract

The vasculature is a dynamic structure, growing and regressing in response to embryonic development, growth, changing physiological demands, wound healing, tumor growth and other stimuli. At the microvascular level, network geometry is not predetermined, but emerges as a result of biological responses of each vessel to the stimuli that it receives. These responses may be summarized as angiogenesis, remodeling and pruning. Previous theoretical simulations have shown how two-dimensional vascular patterns generated by these processes in the mesentery are consistent with experimental observations. During early development of the brain, a mesh-like network of vessels is formed on the surface of the cerebral cortex. This network then forms branches into the cortex, forming a three-dimensional network throughout its thickness. Here, a theoretical model is presented for this process, based on known or hypothesized vascular response mechanisms together with experimentally obtained information on the structure and hemodynamics of the mouse cerebral cortex. According to this model, essential components of the system include sensing of oxygen levels in the midrange of partial pressures and conducted responses in vessel walls that propagate information about metabolic needs of the tissue to upstream segments of the network. The model provides insights into the effects of deficits in vascular response mechanisms, and can be used to generate physiologically realistic microvascular network structures.

## Author summary

During brain development, a two-dimensional mesh-like network of flowing blood vessels spreads over the surface of the growing cortex. Branches from this network penetrate the cortex and connect, forming a three-dimensional network throughout the tissue. Arterio-venous connections on the surface regress, and flow is directed to the interior, so that the cortex is well supplied with oxygen to meet the metabolic demands of neural function. This study aims to better understand how vascular growth, structural remodeling and pruning in response to local metabolic and hemodynamic stimuli generate microvascular networks that meet functional demands. A theoretical model is used to simulate angiogenesis in three dimensions. In this model, a growth factor is generated in tissue at a rate that depends on local oxygen level, and diffuses to existing vessels, where it stimulates increase in diameter and formation of new sprouts if its level is high enough. These mechanisms, along with vascular responses to wall shear stress and pressure, result in networks with similar structural and oxygen transport characteristics to experimentally observed networks. The model shows that vascular responses to metabolic signals, including conducted responses propagated upstream along vessel walls, are essential for formation of adequate network structures. Impairment of conducted responses may contribute to cognitive decline with aging and neurodegenerative diseases.

## Introduction

The effective functioning of the vascular system depends sensitively on its geometrical characteristics. The rate of convective transport by blood depends on the rate of blood flow, which is proportional to the fourth power of vessel diameter according to Poiseuille’s law, for a given pressure gradient and viscosity [1]. Rates of diffusive transport of solutes between blood and surrounding tissue are proportional to the gradients in solute concentration, and therefore vary inversely with the distance that the solute must diffuse. In the case of oxygen transport, the maximum diffusion distance is very short, generally less than 100 μm [2], and so blood flow must pass very close to all parts of the tissue to ensure adequate oxygenation.

This sensitivity of mass transport to geometrical properties implies the need for tight control of vascular network structures in order to meet the functional needs of tissues. The question arises: what are the biological mechanisms that control the geometry of vascular networks? During embryonic development, the locations of the major arteries and veins are under tight genetic control. However, the vascular system contains more than 10^9^ vessel segments, and individual control of their characteristics is clearly not feasible [3]. Moreover, the vasculature is capable of substantial growth and regression during a range of physiological processes such as exercise training, female reproductive cycle, wound healing and tumor growth. These considerations imply that blood vessels must exhibit a set of generic behaviors and responses, enabling them to self-assemble into well-organized networks that meet the needs of the tissue for transport of oxygen and other essential materials [3].

The overall goal of this work is to gain insight into these generic responses. The approach used is to develop biologically plausible hypotheses for the underlying mechanisms, perform theoretical simulations of angiogenesis and structural adaptation based on these hypotheses, and compare the characteristics of the resulting networks with experimental observations. Vascular structures vary greatly with tissue and species. Here, a specific observed network structure in the cerebral cortex of the mouse is used as a basis for comparison [4]. Geometrical, blood flow and oxygen transport properties of this network have been characterized [5], allowing a stringent assessment of results of simulated angiogenesis in this tissue. In previous work [6], we developed a model for angiogenesis in two dimensions based on observations of the rat mesentery, a thin sheet-like tissue. The cerebral cortex vasculature is inherently three-dimensional, and the development of a model for angiogenesis in three dimensions is an aim of the present work.

During the prenatal development of the rodent brain, a vascular plexus spreads over the surface of the neural tissue [7]. These vessels are referred to as the pial vasculature, because they are adjacent to the pia mater, the innermost membrane surrounding the brain tissue. Sprouting angiogenesis from this plexus generates vessels that invade the tissue, forming interconnections and providing perfusion to the developing brain. This begins around embryonic day E9.5 in the mouse [8]. A notable aspect of this developmental process is the necessity for regression and pruning of arterial-venous connections in the surface plexus, which would otherwise shunt blood flow away from the interior of the tissue. In the present model, the initial condition includes a single blood flow pathway on the outer surface of the cortex, and the processes of sprouting angiogenesis, connection of sprouts to form new flow pathways, and pruning of surface arterial-venous connections are simulated.

The development of theoretical models for angiogenesis has been the subject of numerous previous studies. Much of this work has focused on the simulation of angiogenesis in solid tumors [9–21] and wound healing [22–24]. While these are important cases, the high degree of variability in the physiological environment and in the resulting network structures under pathological conditions makes it difficult to calibrate and test the resulting models. With regard to vascular growth in normal tissues, early development by vasculogenesis has been modeled [25,26] but relatively few models have been developed for vascular growth by sprouting angiogenesis in normal tissues [6,27,28]. Angiogenesis in the brain has been considered in the context of models of tumor growth [29] but normal vascular development in the cerebral cortex does not appear to have previously been modeled. The structure and hemodynamics of the mouse cortex microvasculature have been studied in detail [4,5], providing a basis for quantitative assessment of the models presented here.

**Fig 1:**
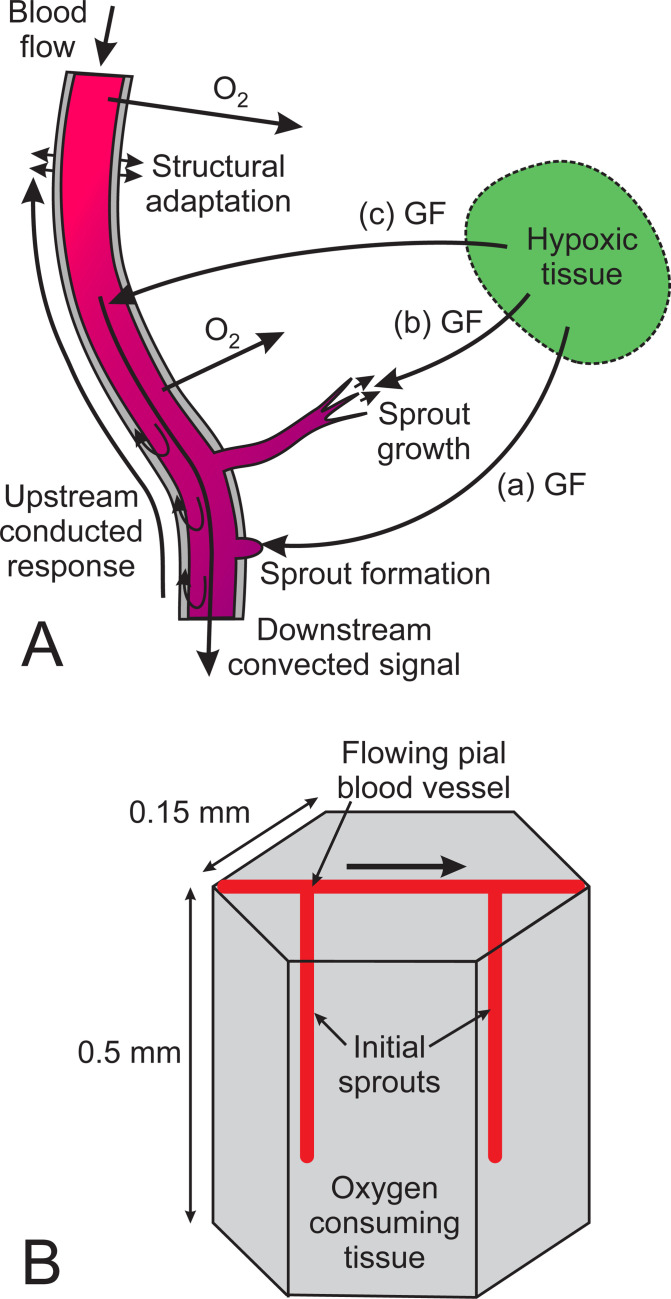
**(A) Schematic representation of assumed mechanisms of vascular growth and adaptation.** Vessels are assumed to respond to local GF concentration in three ways. (a) Existing vessels may develop sprouts if the GF level is above a threshold value. (b) Sprouts elongate with time and connect to other vessels to form flow pathways. The growth direction of each sprout is biased towards regions with higher GF levels. (c) GF reaching a vessel generates a metabolic stimulus that is distributed downstream along the vessel by convection and upstream by conducted responses in vessel walls. (**B**) **Definition of initial configuration.** A hexagonal tissue region is simulated, with periodic boundary conditions in the plane of the hexagon. The region forms part of a repeating structure to represent the cerebral cortex. The initial vascular configuration consists of a single flowing segment representing a pial vessel, and two perpendicular sprouts representing a penetrating arteriole and a penetrating venule. Flow direction in pial vessel is indicated by arrow.

The present approach is based on previously described concepts [6,30,31]. A dynamic model is used to simulate the development of the cortical vasculature. At successive time steps, the spatial distributions of oxygen and a diffusible growth factor are computed, where the production rate of growth factor depends on the local oxygen level. The responses of the vessel network to growth factor are simulated, including angiogenesis, structural adaptation and pruning (Fig 1A). The initial condition consists of a single flowing vessel lying on the surface of the cortex with two non-flowing sprouts (Fig 1B). This model provides insight into the roles that various biological mechanisms play in the generation of stable, functionally adequate network structures, and allows investigation of the effects of downregulating these mechanisms.

## Methods

### Tissue configuration and initial network structure

The simulated region of cortical tissue is a hexagonal prism with height 500 μm and width 300 μm, each side of the hexagon being 150 μm (Fig 1B). The shape is chosen to allow a hexagonally periodic network structure in the plane of the cortex. Any vessel that exits through one side of the hexagon is continued by a corresponding segment entering on the opposite side. Previous work [32] assumed cubic periodicity. The tissue volume (0.0294 mm^3^) is large enough to include a representative vascular network including arterioles, capillaries and venules, while small enough to allow fast computations; computation time increases rapidly with the size of the domain. Parameter values are specified in Table 1.

**Table 1:** Reference parameters.

**Blood oxygen parameters**		**Source**
Maximal RBC oxygen concentration	*C*_0_ = 0.516 cm^3^O_2_ cm^−3^	[36]
Effective oxygen solubility in blood	α_*eff*_ = 3.1 × 10^−5^ cm^3^O_2_ cm^−3^ mmHg^−1^	[36]
Hill equation parameter	*P*_50_ = 40.2 mmHg	[37]
Hill equation parameter	*n* = 2.59	[37]
**Tissue oxygen parameters**		
Krogh diffusion constant	*D*_O*2*_ α = 9.375 × 10^−10^ cm^3^O_2_ cm^−1^ s^−1^ mmHg^−1^	[36]
Consumption rate	*M*_0_ = 7.5 cm^3^O_2_ (100cm^3^) ^−1^ min^−1^	[5]
*P*_*O*2_ at half-maximal consumption	*P*_*c*_ = 10.5 mmHg	[38]
**Growth factor parameters**		
Diffusivity of GF	*D*_*GF*_ = 2 × 10^−7^ cm^2^ s^−1^	*
Tissue GF degradation rate constant	*K*_*GF*_ = 8 × 10^−3^ s^−1^	*
Maximal GF concentration	*C*_*GF*0_ = 1	**
Reference oxygen level for GF release	*P*_*GF*_ = 40 mmHg	*
Exponent for GF release	*N*_*GF*_ = 2.5	*
**Angiogenesis parameters**		
Time step	Δ*t* = 0.05 day	
Diameter of new sprouts	*D*_*s*_ = 8 μm	[39]
Threshold GF concentration for sprouting	*C*_*th*_ = 0.7	*
Constant in sprouting probability function	*C*_*th*50_ = 0.5	*
Maximum sprout formation probability	*k*_*p*_ = 0.22 μm^−1^ day^−1^	*
Sprout growth rate	*V*_*g*_ = 333 μm day^−1^	*
Directional response to GF gradient	*k*_*GF*_ = 20 μm^−1^	*
Attraction constant to nearby vessels	*k*_*V*_ = 1 μm^−1^	*
Maximum vessel sensing distance	*R*_*max*_ = 25 μm	[8]
Maximum vessel sensing angle	θ_*max*_ = π/3	[6]
Variance of growth direction randomization	σ_s_ = 0.05	*
**Vessel migration parameters**		
Threshold for migration	*λ*_*t*_ = 0.25	*
Maximum migration velocity	*v*_*max*_ = 80 μm day^−1^	*
**Structural adaptation parameters**		
Structural adaptation time scale	*T* = 1 day	*
Reference wall shear stress	*τ*_*ref*_ = 0.01 dyn cm^−2^	†
Metabolic sensitivity	*k*_*m*_ = 18	*
Shrinking tendency	*k*_*s*_ = 1.8	*
Vessel permeability to GF (or GF product)	*κ*_*GF*_ = 1	**
Reference flow rate for metabolic signal	*Q*_*ref*_ = 0.1 nl min^−1^	†
Convected response saturation constant	$C_{GF50}^{v}$ = 200 μm	*
Conducted response saturation constant	*J*_*c*50_ = 500 μm	*
Conducted response length constant	*L*_*c*_ = 1.73 cm	[40]
Relative strength of conducted response	$S_{c}^{max}$ = 1	**

*See text for discussion.

**Arbitrary units; set to 1.

†Small constant to avoid singular behavior.

The state of the system is computed at discrete time steps Δ*t*. At each time step, the network is represented as a set of connected straight segments with defined positions, lengths, diameters and blood flow rates. In the initial configuration, blood flow across the upper surface of the tissue is driven by fixed pressures of 100 mmHg at the inflow node and 20 mmHg at the outflow node of a pial vessel. The partial pressure of oxygen (P_O2_) in the inflowing blood is set to 100 mmHg. This vessel includes a narrow upstream segment with fixed diameter of 7 μm and length 35 μm, to represent the flow resistance of upstream pial arteries and arterioles. The resulting initial flow rate is 40.2 nl/min.

A distinctive feature of the cortical vasculature is the arrangement of the penetrating arterioles and venules, which extend into the cortical tissue almost perpendicular to the pial surface [33]. Distal vessels have more random orientations. To incorporate these features in the model, the initial condition includes two straight sprouts extending 400 μm into the tissue perpendicular to the pial vessel (Fig 1B).

### Flow rates

In the simulation of blood flow at each time step, the vessel network is represented as a set of segments connected at nodes [34,35]. Each segment has a flow resistance

$$R=\Delta P/Q=128L\eta_{\mathrm{app}}/(\pi D^{4})$$ (1)

where Δ*P* is pressure drop, *Q* is flow rate, *L* is length, *D* is diameter, and *η*_*app*_ is apparent viscosity of blood, which depends on diameter and hematocrit [1,34]. The condition that the sum of the flows into each internal node is zero yields a set of linear equations for the nodal pressures [41], which is solved iteratively. Effects of phase separation at diverging bifurcations, i.e. unequal partition of hematocrit, are included [1,42]. Because this phenomenon depends on the flow split at each bifurcation, flow resistances depend on flow rates. A further iteration is therefore performed, until flows and hematocrits converge within a specified tolerance. The wall shear stress in each segment is computed as

$$\tau_{w}=D\Delta P/(4L).$$ (2)

### Oxygen transport

At each time step, the transport of oxygen and its spatial distribution in vessels and in tissue are simulated. The oxygen field is assumed to be quasi-steady, and is computed using the Green’s function method [43,44]. In the tissue, the partial pressure of oxygen, P_O2_(*x*,*y*,*z*), satisfies

$$D_{O2}\alpha \nabla^{2}P_{O2}=M(P_{O2})$$ (3)

where *D*_O2_ and *α* are oxygen diffusivity and solubility. Michaelis-Menten kinetics are assumed for oxygen consumption rate:

$$M(P_{O2})=M_{0}P_{O2}/(P_{0}+P_{O2})$$ (4)

where *M*_0_ is oxygen demand and *P*_0_ is P_O2_ at half-maximal consumption. In blood, the convective oxygen flux is

$$f(P_{b})=Q[H_{D}C_{0}S(P_{b})+\alpha_{eff}P_{b}]$$ (5)

where *H*_*D*_ is discharge hematocrit, *C*_0_ is maximal RBC oxygen concentration, α_*eff*_ is effective oxygen solubility in blood,

$$S=P_{b}{}^{n}/(P_{b}{}^{n}+P_{50}{}^{n})$$ (6)

is oxyhemoglobin saturation and *P*_*b*_ is blood P_O2_.

In the Green’s function method, the oxygen field in the tissue is calculated by superposing the fields resulting from arrays of sources and sinks representing oxygen released by vessels and taken up in the tissue. The tissue is discretized as a cubic array of points spaced 20 μm apart, resulting in 3675 points. The vessel network is discretized into segments with lengths averaging about 20 μm, resulting in approximately 1000 segments in a typical simulation. The source strength of a segment is equal to the decrease in *f*(*P*_*b*_) from inlet to outlet. The source and sink strengths are computed using an iterative algorithm such that intravascular and extravascular oxygen levels match at each segment, taking into account the effects of intravascular resistance to radial oxygen diffusion [45].

### Growth factor production and transport

Multiple agents are involved in angiogenesis and structural adaptation of blood vessels, including several forms of vascular endothelial growth factor (VEGF), fibroblast growth factor, tumor necrosis factor-α, transforming growth factor-β, and angiopoietins [46]. These factors have several effects, including stabilization or destabilization of the vessel wall, modulation of wall permeability, formation of sprouts, directional migration of endothelial cells, and differentiation and proliferation of vascular cells. The production of growth factors is sensitive to metabolic conditions in the tissue. In particular, VEGF is released in hypoxic regions, diffuses through tissue, and stimulates growth of new vessels [47,48].

In the present model, a single growth factor (GF) is introduced to represent activities needed for the development of functional vascular networks. Some of the assumed activities align with those of VEGF, but the assumed parameter values are not specific to VEGF. The GF represents in a simplified way the combined actions of the growth factors. It is assumed to be released throughout the tissue depending on the local oxygen level, to diffuse through the tissue and to be degraded with linear kinetics. Its concentration *C*_*GF*_(*x*,*y*,*z*) in tissue satisfies

$$D_{GF}\nabla^{2}C_{GF}=-M_{GF}+K_{GF}C_{GF}.$$ (7)

where *D*_*GF*_ is diffusivity and *K*_*GF*_ is degradation rate. Potential convection of GF by tissue fluid [49] is neglected. The release rate in tissue is assumed to be a decreasing function of local P_O2_:

$$M_{GF}=\frac{K_{GF}C_{GF0}}{1+{\left(P_{O}{}_{2}/P_{GF}\right)}^{N_{GF}}}$$ (8)

Here and elsewhere in the model, Hill-type functions are used to represent biological responses. The constant *P*_*GF*_ is the tissue P_O2_ at half-maximal release rate and the exponent *N*_*GF*_ controls the sensitivity of the response. From these definitions, *C*_*GF*0_ is the maximal GF level that is reached when P_O2_ = 0. The Green’s function method [43] is used to compute *C*_*GF*_ in the tissue domain. The effect of GF uptake by vessels is neglected in the computation of the tissue GF field.

### Sprout formation

The network is assumed to grow by sprouting angiogenesis, represented using a previously developed model [6,14]. At each time step, the GF concentration *C*_*GF*_ at each segment is computed. The assumed probability of sprout formation on that segment is

$$P_{sprout}=\{\begin{array}{l} k_{p}l_{seg}\Delta t\frac{C_{GF}-C_{th}}{C_{th50}+C_{GF}-C_{th}}\mathrm{if}\;C_{GF}>C_{th}\\ 0\;\mathrm{if}\;C_{GF}\leq C_{th} \end{array}$$ (9)

where *k*_*p*_ is the maximal probability of sprout formation per length per time, *l*_*seg*_ is segment length, Δ*t* is the time step, *C*_*th*_ is the threshold level of GF for sprout formation and *C*_*th*50_ determines the GF level for half-maximal sprout probability. The location of a new sprout on the parent segment is chosen randomly. If it is within 10 μm of a node, it is moved to that node, and if it is at a network boundary node or an existing branch point, it is suppressed. These rules are needed to avoid formation of very short segments and other anomalous structures. The direction of sprouting is chosen at random in the plane perpendicular to the parent segment.

### Elongation of sprouts

Sprouts grow at a rate *V*_*g*_ and maintain a fixed diameter *D*_*new*_ until they connect with another vessel. The growth direction varies at each time step in response to three types of input: (i) random variation; (ii) “homing” to other segments; (iii) responses to GF gradients. These effects are implemented as follows. (i) To simulate the stochastic nature of vessel growth, the growth direction ***d*** at the previous step is rotated through a random angle in three dimensions with variance σ_s_, giving a new direction ***d****’*. (ii) To allow growing sprouts to detect and connect to other segments, a homing mechanism is introduced, representing the activity of the filopodia of the tip cells leading sprout growth, which explore the tissue ahead of the sprout and may sense other vessels [50]. A conical region is defined that extends a distance *R*_*max*_ from the tip at angles up to *θ*_*max*_ from the direction ***d****’*. A vector directed towards segments within this region is defined:

$$d_{V}=\sum \int e_{r}f(r)g(\theta )\;ds$$ (10)

where the sum is over the segments within the sector, the integral is along each segment, **e**_*r*_ is a unit vector directed from the tip to the segment, *r* and *θ* describe the positions of points on each segment relative to the current tip position and orientation, and *f* and *g* are chosen to give a weighting factor that decays to zero at the edges of the region:

$$f(r)=\{\begin{array}{l} 1-r/R_{max}\;\mathrm{if}\;r<R_{max}\\ 0\;\mathrm{if}\;r\geq R_{max} \end{array},$$ (11)

$$g(\theta )=\{\begin{array}{l} 1-\frac{1-\cos\theta }{1-\cos\theta_{max}}\mathrm{if}\;\theta <\theta_{max}\\ 0\;\mathrm{if}\;\theta \geq \theta_{max} \end{array}.$$ (12)

(iii) To represent the tendency of sprouts to grow up gradients of GF [14], the vector gradient ∇*C*_*GF*_ at the sprout tip is computed. The updated sprout direction is

$$d”=d\prime +k_{V}d_{V}+k_{GF}\nabla C_{GF}$$ (13)

where *k*_*V*_ and *k*_*GF*_ represent sensitivity of growth direction to existing vessels and to GF gradients.

During one time step, each sprout grows a distance *V*_*g*_Δ*t*, This is done in increments of 5 μm, and a connection is created if the tip comes within 5 μm of another segment. Other adjustments are made as needed to avoid anomalous structures [6].

### Tension-induced migration

The formation of sprouts as described above results in bifurcations with branching angles of 180° and 90°, whereas observed networks show a smooth distribution of branching angles clustered around 120°. This discrepancy implies that vessels must migrate through tissue after bifurcations form [6]. Blood vessels are under longitudinal tension *in vivo* [51,52] and collagen and other interstitial components are subject to turnover [53], so that vessels may migrate through the interstitium. To simulate this, the normalized force acting on each node in the network is computed, assuming that the tension in each vessel is proportional to diameter:

$$f_{t}=\left(\sum D_{i}e_{i})(\sum D_{i}\right)/\left(\sum l_{i}D_{i}\right)$$ (14)

where the sum is over the segments at the node, *D*_*i*_ are diameters, *l*_*i*_ are lengths and **e**_*i*_ are unit vectors parallel to the segments. This is a modified version of the previous model [6]. At an unbranched node, |**f**_*t*_| gives a dimensionless estimate of vessel curvature. If |**f**_*t*_| exceeds a threshold *λ*_*t*_, the node migrates with velocity

$$v=v_{\max}f_{t}(1-\lambda_{t}/|f_{t}|)$$ (15)

where *v*_*max*_ is the maximum speed and *λ*_*t*_ defines the ratio of vessel diameter to threshold radius of curvature. The threshold allows stabilization of curved vessels, which would otherwise eventually straighten out.

### Structural adaptation and pruning

Structural adaptation of vessel diameters is essential for the generation of functional and efficient vascular networks [6]. The present model follows previous work [40,54], but with modifications to achieve stable network structures in three dimensions. It is based on the assumption that structural change of vessel diameters occurs in response to the combined effects of four types of signals: wall shear stress *τ*_*w*_, intravascular pressure *P*, downstream convected metabolic signal *S*_*m*_ and upstream conducted metabolic signal *S*_*c*_. Each segment diameter *D* thus varies at each time step according to

$$\Delta D=S_{tot}D\Delta t/T$$ (16)

where Δ*t* is the time step, *T* is a characteristic timescale, and *S*_*tot*_ is the dimensionless total adaptive signal:

$$S_{tot}=log(\tau_{w}+\tau_{ref})-\log\;\tau_{e}(P)+k_{m}(S_{m}+S_{c})/D-k_{s}.$$ (17)

The logarithmic dependence on *τ*_*w*_ is included to ensure sensitivity over the observed wide range of wall shear stresses [55], including the tendency of segments experiencing low levels of wall shear stress to regress [56]. The small constant *τ*_*ref*_ is included to avoid singular behavior. The correlation of the expected shear stress *τ*_*e*_ (in dyn/cm^2^) with *P* (in mmHg) previously reported [40] is here fitted using a Hill-type equation

$$\tau_{e}(P)=14+86\frac{P^{5}}{P^{5}+P_{\tau }^{5}}$$ (18)

with *P*_*τ*_ = 36 mmHg. The present model differs from that used previously [6] in that the contributions of *S*_*c*_ and *S*_*m*_ to *S*_*tot*_ are assumed to vary inversely with *D* in Eq (17). Such dependence was necessary to avoid excessive dropout of small vessels while also avoiding unrealistically large diameters of main feeding and draining vessels.

Adaptive responses to metabolic needs are represented by the dimensionless signals *S*_*m*_ and *S*_*c*_, both of which depend indirectly on tissue oxygen levels, via GF that is generated in the tissue and diffuses to vessels. The GF may enter vessels, where it is convected downstream, or it may stimulate release of other signaling substances by endothelial cells in proportion to GF level. The model is applicable to either situation. In the previous model [6], metabolic signals depended on intravascular oxygen levels, whereas in the present model, metabolic signals are generated in the tissue [57] and transmitted to the vessels by diffusion of GF (Fig 1A).

The steps in computing *S*_*m*_ and *S*_*c*_ in each segment are as follows. (i) Under the assumption that GF diffuses into each segment at a rate proportional *C*_*GF*_, the convective flux of GF is given by

$$\frac{dJ_{GF}}{ds}=\kappa_{GF}C_{GF}$$ (19)

where *C*_*GF*_ is the concentration in the adjacent tissue, *κ*_*GF*_ is a permeability coefficient and *s* is downstream distance along each segment. The flux is initialized to zero at network inflows. (ii) The vessel GF concentration is

$$C_{GF}^{v}=\frac{J_{GF}}{Q+Q_{ref}}$$ (20)

where *J*_*GF*_ is evaluated at the midpoint of the segment, *Q* is flow rate in nl/min and *Q*_*ref*_ is a small constant to avoid singular behavior. (iii) The downstream convected metabolic signal is a function of concentration:

$$S_{m}=\frac{C_{GF}^{v}}{C_{GF}^{v}+C_{GF50}^{v}}$$ (21)

(iv) The convected metabolic signal stimulates an upstream conducted response in the vessel wall, denoted *J*_*c*_, which decays with distance traveled:

$$\frac{dJ_{c}}{ds}=S_{m}-J_{c}/L_{c}$$ (22)

where *s* here denotes upstream distance. The signal is initialized to zero at network outflows. At converging bifurcations relative to direction of conduction, incoming signals are weighted by the diameters of the vessels and summed. At diverging bifurcations, the outgoing signal is divided among the upstream vessels in proportion to their diameters. (v) The conducted metabolic signal is

$$S_{c}=S_{c}^{max}\frac{J_{c}}{J_{c}+J_{c50}}$$ (23)

where $S_{c}^{max}$ controls the relative magnitudes of convected and conducted signals.

Pruning of redundant vessels is simulated by assuming that a vessel drops out if its diameter drops below 3 μm, the approximate minimum for passage of red blood cells [58]. Any other segments whose flows cease as a result are also pruned.

### Parameter values

Table 1 gives parameter values. Precise values are stated for computational reproducibility, but the number of decimals shown does not imply a corresponding precision in their estimates. Oxygen transport parameter values for blood and tissue are from previous studies [5,36,37]. Parameter values for GF transport, angiogenesis and structural adaptation are not generally available *a priori*. A feasible set of values (the reference parameter set) was obtained by performing many simulations to explore the parameter space and applying the following criteria. (i) The simulation converges to a stable final state within the simulation period of 10 days, which represents the time for development of cortical microcirculation [7]. Stability requires that *C*_*GF*_ < *C*_*th*_ throughout the domain, so that no further sprouts are formed. (ii) In the final state, tissue P_O2_ > 20 mmHg throughout the domain [5]. (iii) The initial A-V connection on the pial surface is pruned [7]. (iv) The final vessel length density matches that in the experimental network [5]. (v) The final oxygen extraction matches that computed for the experimental network [5]. An objective function was defined as:

$$E=F_{GF}{}^{2}+F_{H}{}^{2}+{\left(L_{t}-L_{0}\right)}^{2}+(E_{OX}-E_{OX}{}_{0}{)}^{2}$$ (24)

where *F*_*GF*_ and *F*_*H*_ are the percent of tissue points with *C*_*GF*_ > *C*_*th*_ and P_O2_ < 20 mmHg respectively, *L*_*t*_ and *E*_*OX*_ are the total vessel length in mm and the percent oxygen extraction, and *L*_0_ = 19.2 and *E*_*OX*_ = 26.2 are the target values based on experimental data [5]. Minimization of this objective function strongly restricts allowable parameter sets, but the available experimental data do not permit unique identification of the unknown model parameters.

Some parameters can be fixed without loss of generality. GF concentrations are stated in arbitrary units, and the maximal concentration *C*_*GF*0_ is set equal to 1. The model results depend on the ratio of the diffusion constant *D*_*GF*_ and the degradation rate *K*_*GF*_, and not on their individual values. The length scale defined by *L*_*GF*_ = (*D*_*GF*_/*K*_*GF*_)^1/2^ must be large enough that vessels are responsive to nearby hypoxic tissue, but short enough that vascular responses are localized to the region of hypoxia. The reference parameter values correspond to *L*_*GF*_ = 50 μm. Because *J*_*GF*_ in Eq (19) is expressed in arbitrary units, *κ*_*GF*_ is set to 1.

A time step of 0.05 day is used for simulations. The maximal probability of sprout formation (0.22 μm^−1^ day^−1^) gives a probability less than 0.22 for sprout formation in each time step by a 20-μm segment. The assumed growth rate of sprouts corresponds to 16.65 μm per time step, and the maximum rate of migration of nodes due to vessel tension corresponds to 4 μm per time step. The characteristic time scale for structural adaptation (Eq (16)) is set to *T* = 1 day. This gives faster remodeling than the value of 4.5 days estimated for remodeling in the adult mouse [59]. These parameters values result in development of a stable network in about 5 days, comparable to the rapid development of the cerebral microcirculation in the prenatal mouse. However, the assumed timescale is not inherent in the model. Identical results on a different timescale would be obtained with a simple rescaling of the abovementioned parameters.

The threshold concentration for sprout formation is a critical parameter. If it is too low, network structures are unstable due to GF levels above threshold. If it is too high, tissue is not well oxygenated. The effects of varying this parameter are discussed below. The maximum vessel sensing distance (*R*_*max*_ = 25 μm) is similar to the observed length of endothelial tip cell filopodia in mouse brain [8]. The attraction constant for tip cells to nearby vessels is set to a high value (*K*_*V*_ = 1 μm^−1^) to ensure efficient formation of connections. The threshold (*λ*_*t*_) for vessel migration gives an equilibrium radius of curvature of 40 μm for a 6-μm diameter vessel.

### Sensitivity analysis

The sensitivity of three key output variables (total vessel length, total blood flow rate and oxygen extraction) to the values of several model parameters was analyzed. Each parameter was varied to values above and below its reference value, by increments of between 5% and 50%. The sensitivity of output variable *X* on parameter *p* is defined as (Δ*X*/*X*_*ref*_) / (Δ*p*/*p*_*ref*_), where Δ*p* is the difference between the high and low tested values of the parameter, Δ*X* is the corresponding change in the output variable, and *p*_*ref*_ and *X*_*ref*_ are the values under reference conditions.

### Computational procedures

The simulation is implemented using the C++ language on personal computers. The most computationally demanding aspect is the calculation of the oxygen and GF fields at each time step using the Green’s function method. A system with graphical processor units (Pascal Quadro GP100, NVIDIA, Santa Clara, CA) was used to accelerate this computation. Due to the randomization of sprout formation and growth direction, the simulation results vary with the seed used for the random number generator. For reported quantitative results, simulations with 8 seeds are averaged, except where otherwise noted. The execution time per time step varies from about 1 s to several minutes depending on the network complexity. A simulation with 200 time steps takes 2 hours or more. Thousands of such simulations were performed in the process of developing and refining the model. Computer codes and data files are available at https://github.com/secomb.

## Results

**Fig 2:**
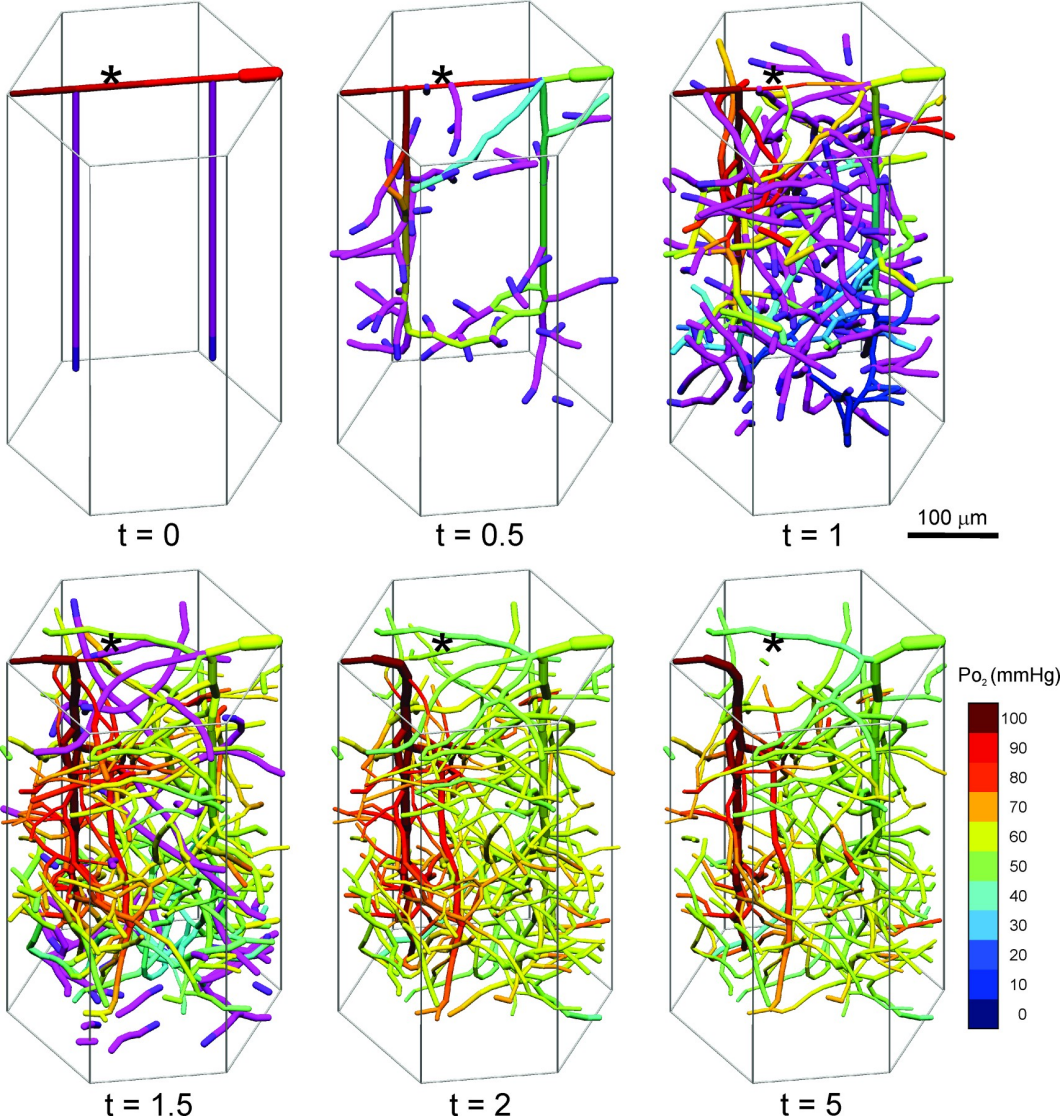
Example of simulated angiogenesis, remodeling and pruning. Network structure within a single hexagonal unit is shown at several time points, indicated in days. Flowing vessels are color-coded by intravascular oxygen level according to the color bar. Non-flowing sprouts are shown as purple, with tips shown as dark purple. Apparently disconnected segments are actually connected via adjacent hexagonal units. Asterisk (*) shows position of initial pial connection between arteriole and venule, illustrating shrinkage and eventual pruning of this connection, such that flow is redirected to the interior of the cortical tissue.

An example of simulated network development is illustrated in Fig 2. During the initial phase, non-flowing sprouts branch off the preexisting arteriole and venule. The first connections between the arteriole and venule (A-V) form at about 0.5 days and start to flow. The A-V connection on the pial surface shrinks and is pruned by 1.5 days. The length of vessels in the domain reaches a maximum at about 1.5 days, declining thereafter due to pruning. A steady state of the network is achieved by 5 days.

**Fig 3:**
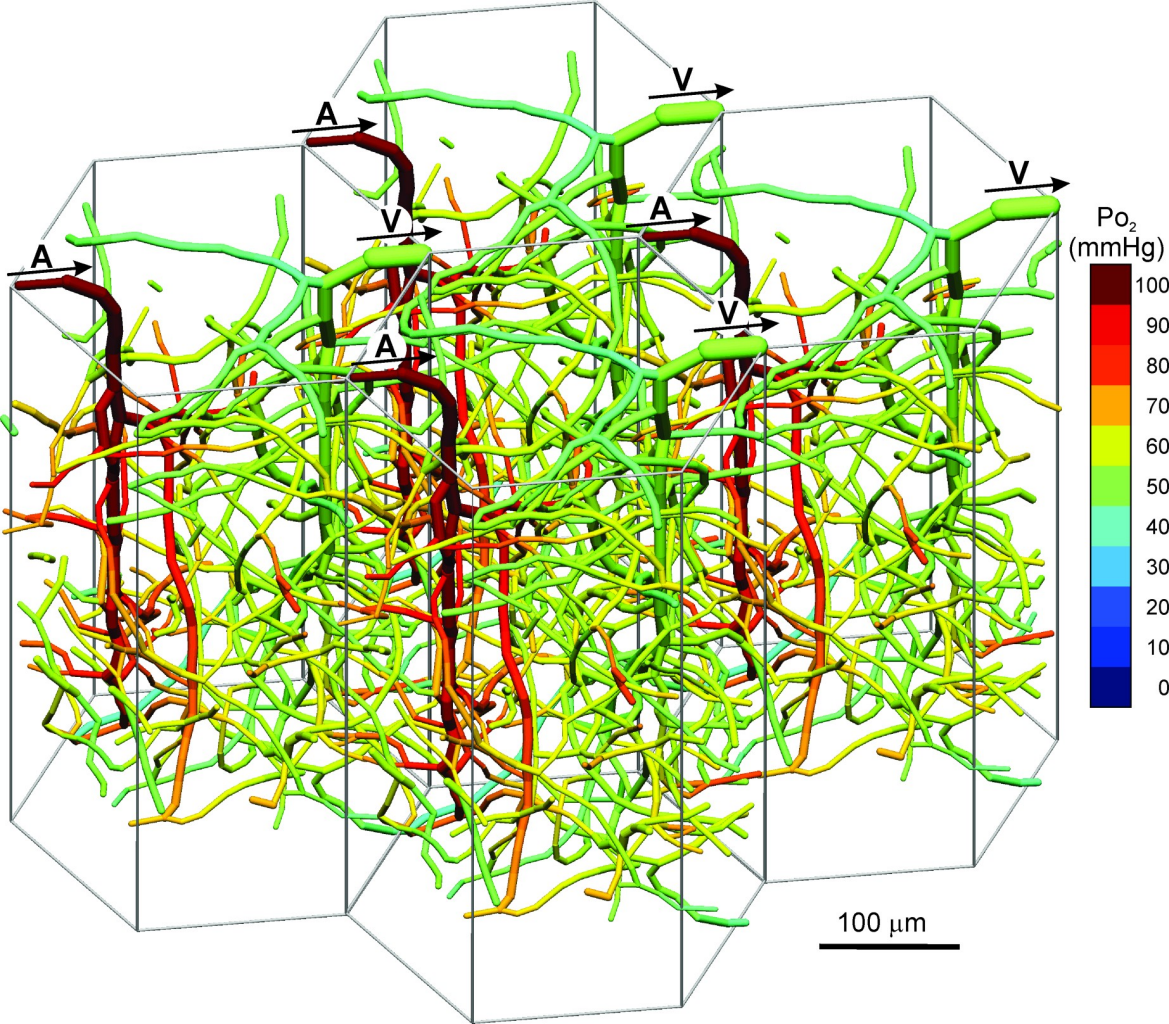
Final vessel configuration. Four adjacent identical hexagonal units are illustrated, to show continuity of network structure across boundaries of the hexagon. A: Penetrating arteriole. V: Penetrating venule. Arrows indicate flow direction.

To give a more complete view of the network geometry, four replicates of the domain are combined in Fig 3. Numerous vessels cross the boundaries of each domain, forming a continuous mesh-like network that extends throughout the cortical layer. This structure appears similar to observed networks in the cortex of mouse [5,60] and human [33,61].

**Fig 4:**
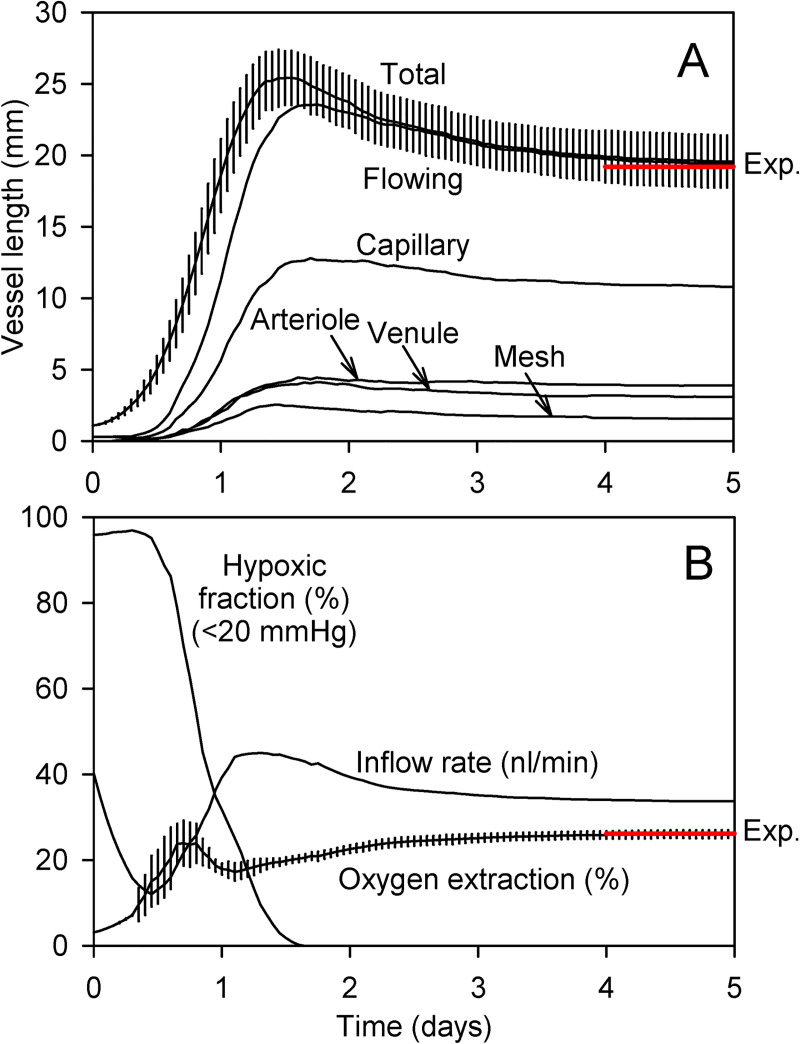
Dynamics of network generation. Results are the average of 8 simulations with different initial seeds for the random number generator. Vertical bars show ±1 standard deviation. **(A)** Vessel length. Total length includes flowing vessels and non-flowing sprouts. Flowing vessels are further classified as arterioles capillaries, venules and mesh segments. Red line (Exp.): Expected vessel length based on experimentally observed length density in mouse brain cortex [5]. **(B)** Flow and oxygen transport parameters. Results for blood flow rate to the network, oxygen extraction and hypoxic fraction are shown. Red line (Exp.): Expected extraction based on simulations of oxygen transport in an experimentally observed network [5].

Fig 4A shows the time course of parameters describing vessel length in the simulated networks. The total length of flowing vessels starts to increase at about 0.5 days, due to the formation of A-V connections, reaching a maximum and then declining to approach a steady state. Model parameters were adjusted so that the final total length (averaged over 8 runs) corresponds to a vascular density of 658 mm^−2^, close to the experimental value [5]. Flowing vessel segments are classified according to the types of bifurcations at their upstream and downstream ends, as “arteriolar” (diverging to diverging), “venular” (converging to converging), “capillary” (diverging to converging) and “mesh” (converging to diverging) [55]. The total lengths of all types of flowing segments reach maximal values at about 1.5 days and then decline. At steady state, mesh segments then represent about 8% of the total vascular network. Although the network appears mesh-like, this statistic indicates that the structure consists mainly of a diverging arteriolar tree connected by capillaries to a converging venular tree.

Fig 4B shows the behavior of parameters describing blood flow and oxygen transport. Blood flow rate increases initially, reaching a maximum at about 1 day, and then declines slightly. The hypoxic fraction of the tissue is defined as the fraction of tissue points with P_O2_ < 20 mmHg. This criterion is chosen to reflect the sensitivity of neural tissue to even moderate hypoxia. In the initial configuration, nearly all the tissue is hypoxic. When A-V connections start to form at 0.5 days, the hypoxic fraction declines, reaching zero at about 1.5 days. Oxygen extraction is defined as the A-V decrease of oxygen concentration in blood as a percent of the concentration in the inflowing arteriole. This quantity fluctuates during the initial phase, then approaches 26.27 ± 1.06% (mean ± s.d). Model parameters were adjusted to give agreement with the value 26.2% obtained from oxygen transport simulations for the experimentally observed network [5].

Quantitative comparisons of simulated network properties with those of the experimentally observed network [5] are presented in Table 2. The perfusion (flow per volume) values match closely, as a consequence of matching oxygen extraction values and assuming the same oxygen demand. The distribution of distances of tissue points to the nearest flowing vessel strongly influences the distribution of oxygen levels. As indicated by Table 2, the simulated networks have broader distributions of this parameter (i.e. higher mean and s.d.) than the observed network, despite having the same vascular density. This may be a consequence of the relatively sparse network in the lower part of the domain (Fig 3), which results from the lack of connections to deeper vasculature. Average vessel and tissue oxygen levels are slightly higher for the simulated networks than for the observed network. Overall, the oxygen transport characteristics of the simulated networks agree approximately with those of the observed network [5].

**Table 2:** Comparison of simulated networks with experimentally observed network properties.

	Observed network [5]	Simulated networks: mean ± s.d. (n = 8)
*Total vessel length density (mm^−2^)	658.6	658.0 ± 64.8
*Oxygen extraction (%)	26.2	26.27 ± 1.06
Effective perfusion (cm^3^ (100cm^3^)^−1^ min^−1^)	110.4	114.5 ± 5.3
Mean distance of tissue points to nearest vessel (μm)	15.061	19.04 ± 1.51
S.d. of distance of tissue points to nearest vessel (μm)	9.365	13.44 ± 1.99
Mean vessel P_O2_ (mmHg)	54.53	61.02 ± 0.56
S.d. of vessel P_O2_ (mmHg)	14.07	11.16 ± 0.55
Mean tissue P_O2_ (mmHg)	47.28	49.81 ± 1.76
S.d. of tissue P_O2_ (mmHg)	12.17	9.32 ± 0.84

*The model parameters were adjusted to fit these two model outputs to values for the observed network

Results of the sensitivity analysis are shown in Table 3. Because oxygen extraction varies inversely with flow rate for a given oxygen consumption rate, extraction and flow rate have approximately opposite sensitivities to each parameter. Total vessel length shows a different pattern of parameter sensitivities, with highest sensitivity to the GF threshold for sprouting (*C*_*th*_). The value of this parameter was determined as follows. For *C*_*th*_ values below 0.6, regions with GF above threshold persisted and stable networks could not be achieved. Conversely, values above 0.8 led to persistent tissue hypoxia (P_O2_ < 20 mmHg). An intermediate value of 0.7 was assumed.

**Table 3:** Sensitivities of total vessel length, total flow and oxygen extraction to model parameters.

Parameter	Vessel length	Total flow	Extrac-tion
*C*_*th*_, threshold GF concentration for sprouting	**−1.080**	−0.432	+0.369
*k*_*p*_, maximum sprout formation probability	+0.384	+0.173	−0.155
*k*_*GF*_, directional response to GF gradient	−0.009	−0.004	+0.006
*P*_*GF*_, reference oxygen level for GF release	**+0.703**	**+1.054**	**−0.962**
*N*_*GF*_, exponent for GF release	+0.130	−0.143	+0.144
*λ*_*t*_, threshold for migration	+0.171	+0.104	−0.106
*k*_*m*_, metabolic sensitivity	−0.222	**+0.948**	**−0.888**
*k*_*s*_, shrinking tendency	+0.364	**−1.245**	**+1.212**
$C_{GF50}^{v}$, convected response saturation constant	−0.025	−0.549	+0.499
*J*_*c*50_, conducted response saturation constant	−0.097	−0.282	+0.253
$S_{c}^{max}$, relative strength of conducted response	−0.281	+0.422	−0.416

Sensitivity values with magnitude greater than 0.7 are shown **bold**.

Two parameters whose values sensitively influence model results are the reference oxygen level for GF release (*P*_*GF*_) and the overall metabolic sensitivity (*k*_*m*_), indicating that responses to tissue metabolic status are an important component of the model. Previous studies indicate that tissue oxygen levels in the cortex under normal conditions are generally above 20 mmHg [5,62]. For the model to give this behavior, GF production rate must be sensitive to oxygen levels well above 20 mmHg. A relatively high value for the tissue P_O2_ for half-maximal GF release is therefore required, and *P*_*GF*_ = 40 mmHg is assumed in the reference parameter set.

The results are insensitive to the value of *k*_*GF*_, which defines the directional response of sprout growth to GF gradient. Directional guidance of sprout growth by VEGF gradients has been considered an important factor in angiogenesis [14], but the current simulations do not suggest a role for such guidance in the vascularization of the brain cortex.

To examine the role of metabolic responses in angiogenesis, further simulations were performed with reduced values of *k*_*m*_, which determines the metabolic response including convected (downstream) and conducted (upstream) responses, and *S*_*c*_^*max*^, which determines the strength of the conducted response relative to the convected response. As indicated in Table 3, decreasing these parameters reduces total blood flow. To compensate for this, the parameter *k*_*s*_ giving the shrinking tendency of vessel diameters was adjusted in each case, so that the flow at the end of each run was within 8% of the reference value. Maintaining total flow in this way allowed assessment of the role of metabolic responses in determining the distribution of flow in the region.

**Fig 5:**
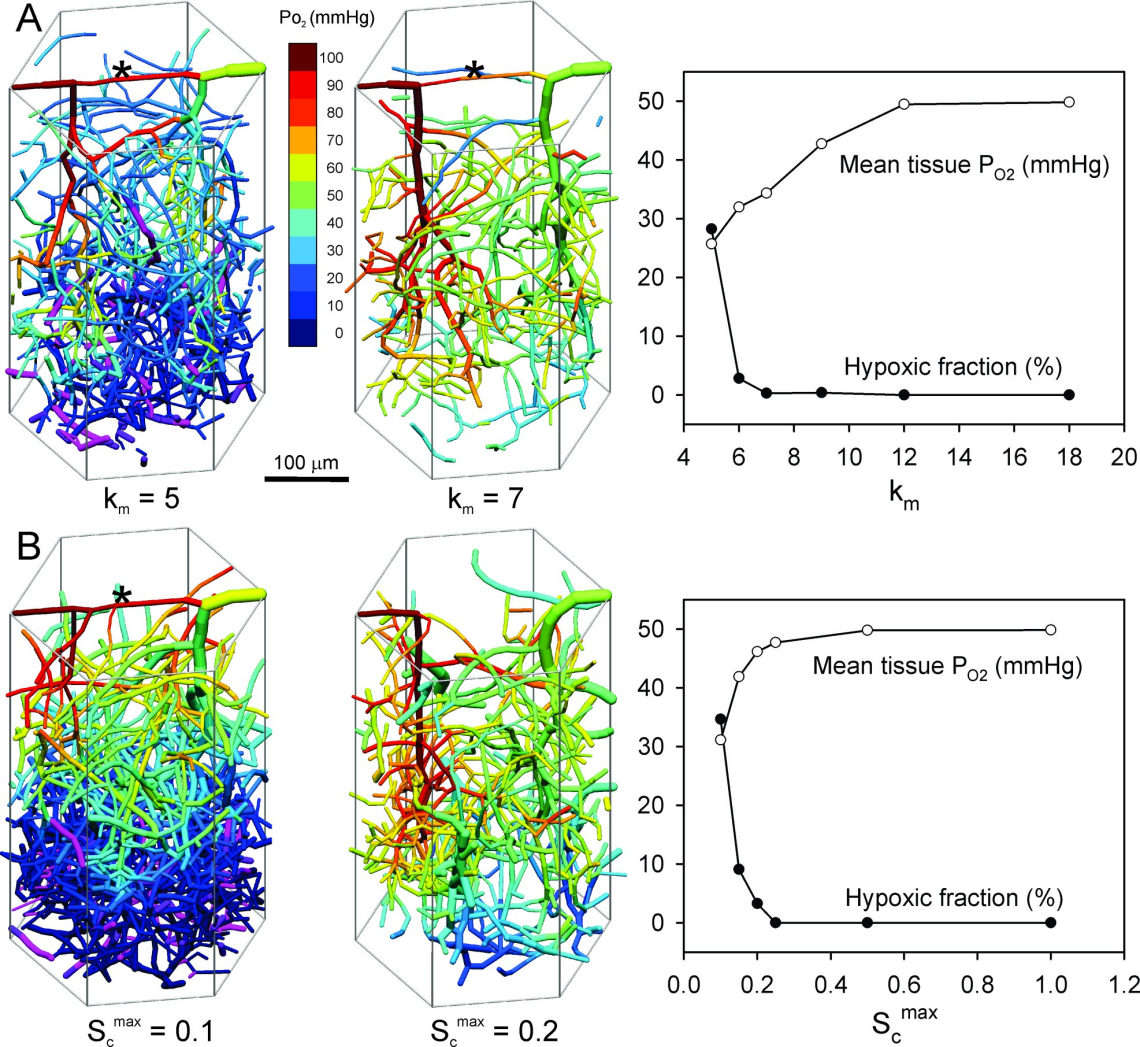
Effects of reduced metabolic responses. Graphs show values of mean tissue P_O2_ and hypoxic fraction at end of simulation at 10 days (2 days for the case *S*_*c*_^*max*^ = 0.1). Examples of final network structures, color-coded for vessel P_O2_, are shown at left of each graph. Asterisk (*) indicates retention of pial A-V connection. **(A)** Effect of reducing strength of metabolic response (*k*_*m*_) while holding total flow nearly constant. **(B)** Effect of reducing relative strength of upstream conducted response (*S*_*c*_^*max*^) while holding total flow nearly constant.

Fig 5A illustrates the effects of decreasing the metabolic sensitivity *k*_*m*_ from its reference value (*k*_*m*_ = 18). These results are for a single run at each parameter setting. When *k*_*m*_ decreases below 12, mean tissue P_O2_ declines. When *k*_*m*_ ≤ 7, the pial A-V connection is not pruned, and oxygenated blood is shunted away from the underlying cortical tissue. The tissue hypoxic fraction rises sharply, and network structure does not approach a stable state. These simulations show that a structural response to metabolic signals is necessary in order to generate network structures that provide adequate tissue oxygenation to brain cortex.

The effects of decreasing *S*_*c*_^*max*^ from its reference value (*S*_*c*_^*max*^ = 1) are shown in Fig 5B. The resulting behavior is similar to that when *k*_*m*_ is reduced. The pial A-V connection is retained, mean tissue P_O2_ declines, and network structure is unstable. According to these results, local and downstream convected metabolic responses are not able to maintain adequate network structures. Conducted responses propagated upstream along arterioles are essential for maintaining tissue oxygenation, as shown previously for mesenteric networks [54].

## Discussion

The generation of functional microvascular networks requires angiogenesis, remodeling and pruning, which generally occur in parallel [63]. In previous work [6], we demonstrated theoretically how these processes, occurring in response to local metabolic and hemodynamic signals, can generate stable, functionally adequate structures in two dimensions, with properties matching those of observed microvascular networks in the mesentery. The present work extends the previous approach to a three-dimensional microvascular network, the vasculature of the brain cortex.

The criteria used to determine the reference parameters restrict their values but do not uniquely define them. As shown in Table 3, parameters have overlapping effects on key output variables, and equivalent results could be obtained using other combinations of parameter values. The reference parameters for angiogenesis and structural adaptation do not represent accurate estimates of actual biological effects. Indeed, such effects can be assumed to have significant natural variability. However, the objective of this work is not to determine values of parameters describing angiogenesis and structural adaptation, but rather to demonstrate that networks with characteristics matching those of observed networks can be simulated by a theoretical model including the assumed set of biologically plausible mechanisms.

In the early development of the cortical microcirculation, a notable feature is the transition from a nearly two-dimensional network on the pial surface to a three-dimensional network extending through the cortical layer [7]. The present model simulates this behavior, including the loss of A-V connections on the pial surface that would otherwise shunt blood away from the interior of the cortex. The model shows the development of a continuous mesh-like network of capillaries within the cortical layer, similar to observed microvascular structures. The hexagonally periodic geometry allows the model to represent this continuous network structure (Fig 3).

The simulations permit investigation of the roles played by various response mechanisms, by varying the parameters controlling the strengths of these responses. The results confirm the importance of responses to metabolic signals in order to ensure that tissue is adequately supplied with blood flow [6]. Downregulation of these responses while maintaining overall perfusion results in impaired flow to the deeper part of the cortex, with the development of hypoxia (Fig 5A). Contributing to this behavior is the retention of the pial A-V connection under this condition. Similar behavior is predicted when only the upstream conducted response is downregulated (Fig 5B). This result indicates the essential role of conducted responses in maintaining functionally adequate network structures in the brain.

Experimental evidence supports the concept that conducted responses play an important role in the maintenance of vascular structures throughout the body. The role of conducted responses in coordinating acute vasomotor responses (i.e. flow regulation) is well established [64]. Conducted responses generated in venules have been observed to traverse the capillary bed and affect vascular tone in upstream arterioles [65]. Furthermore, chronic changes in vascular tone have been shown to lead to corresponding changes in structural vessel diameters [66,67], which implies a role for conducted responses in structural remodeling. Conducted responses are transmitted via gap junctions that connect endothelial cells. Genetic ablation of gap junction proteins in mice has been shown to affect vascular structures [68,69], a finding consistent with an essential role for conducted responses in structural adaptation.

Theoretical simulations are used to study various aspects of microcirculatory function in the brain, such as neurovascular coupling [70]. Some of these studies have been based on computer-generated synthetic capillary network structures [32,71,72], because structural information at capillary scales is difficult to obtain experimentally. Generally, these synthetic structures have been shown to be consistent with experimentally observed networks with regard to parameters such as vessel diameter and length, and surface and length densities. However, the approach presented here has advantages over previous methods for producing synthetic microvascular structures. (i) Networks are generated by algorithms that simulate known mechanisms of angiogenesis. (ii) Networks include hierarchical, tree-like arteriolar and venular structures in addition to capillaries. (iii) Blood flow and oxygen transport characteristics match predictions based on observed network structures in the mouse cortex.

The vascular component of neurodegenerative diseases is increasingly recognized but remains poorly understood [73]. Development of hypoxic micro-regions may contribute to cognitive decline in elderly people [74]. We previously proposed that the highly heterogeneous tissue oxygenation typically observed in solid tumors results from impaired conducted responses [75]. Because conducted responses depend on intact gap junctions between endothelial cells forming a continuous pathway for propagation of signals, they may be vulnerable if endothelial cells are dysfunctional. The results of the present model support the concept that reduction in conducted responses in age or disease may contribute to cognitive impairment in humans.
